# A comparison of real-time PCR and reverse line blot hybridization in detecting feline haemoplasmas of domestic cats and an analysis of risk factors associated with haemoplasma infections

**DOI:** 10.1186/1746-6148-8-103

**Published:** 2012-07-02

**Authors:** Karla Georges, Chuckwudozi Ezeokoli, Tennille Auguste, Nisshi Seepersad, Akua Pottinger, Olivier Sparagano, Séverine Tasker

**Affiliations:** 1 School of Veterinary Medicine, Faculty of Medical Sciences, The University of the West Indies, St Augustine Campus, Eastern Main Road, St. Augustine, Trinidad and Tobago, , West Indies; 2 University of Agriculture, College of Veterinary Medicine, Makurdi, Nigeria; 3 School of Health Community and Education Studies, Northumbria University, Coach Lane Campus, Newcastle upon Tyne, NE7 7XA, UK; 4 School of Veterinary Sciences, University of Bristol, Langford, Bristol, UK

**Keywords:** Feline haemoplasmas, qPCR, Reverse line blot, Feline retrovirus infection

## Abstract

**Background:**

Three species of feline haemoplasma are recognised: *Mycoplasma haemofelis* (Mhf), ‘*Candidatus* Mycoplasma haemominutum’ (CMhm) and ‘*Candidatus* Mycoplasma turicensis (CMt). This study compared a reverse line blot hybridization (RLB) assay for simultaneous detection of Mhf, CMhm with three separate quantitative real-time polymerase chain reaction (qPCR) assays used for diagnosis of Mhf, CMhm and CMt. The RLB and qPCR assays were applied to DNA extracted from blood samples collected from 154 cats from Trinidad and Tobago.

**Results:**

CMhm and Mhf DNA were detected using both RLB and qPCR. CMt DNA was detected by qPCR only. Comparing RLB and qPCR for the detection of CMhm DNA, 40 (26.3%) and 48 (31.6%) cats, respectively, were positive. The difference was more marked for Mhf, with RLB detecting a total of only 11 (7.2%) positive cats whereas qPCR detected 41 (27.0%) positive cats. Using qPCR as a gold standard, haemoplasma infected cats were more likely to be retrovirus positive (OR = 5.68, P = 0.02) and older (median age 5.5 years), than non-infected cats. In addition, CMhm positive cats were more likely to be male (OR = 3.4, P = 0.04).

**Conclusions:**

Overall the qPCR was more sensitive than RLB. In addition, age (median 5.5 years) and retrovirus positivity were risk factors for infection with the feline haemoplasmas in this study population. Further studies on feline haemoplasma infections in cats are needed to determine the significance of detecting small amounts of haemoplasma DNA, feline retrovirus infection and other associated risk factors on the clinical manifestation of disease.

## Background

Feline infectious anaemia is caused by haemotropic bacteria of the genus *Mycoplasma*. These agents, formerly named *Haemobartonella* spp, are now reclassified to the genus *Mycoplasma* based on the 16S rRNA gene sequences [1]. They have also been given the trivial name of haemoplasmas [1]. Three species of feline haemoplasmas are recognised: ‘*Candidatus* Mycoplasma haemominutum’ (CMhm), *Mycoplasma haemofelis* (Mhf) and ‘*Candidatus* Mycoplasma turicensis (CMt). All three haemoplasmas are known to have a worldwide distribution [2-5]. Infections with feline haemoplasmas may be asymptomatic, or can lead to anorexia, lethargy, acute haemolysis and sudden death. Recovery from clinical disease may be followed by a carrier state [6]. Data from experimental infections have demonstrated Mhf to be more pathogenic than CMhm [7,8]. However, infection with CMt in experimental cats has resulted in variable pathogenicity [9,10], and its pathogenic potential probably depends on several agent cofactors [11].

Traditionally, haemoplasma infections were diagnosed through cytological examination of blood smears but this method is known to be insensitive and cannot differentiate between the different haemoplasma species [7,12]. Sensitive PCR techniques are now used [13-15] with quantitative (q) PCR assays able to detect and quantify organisms [4,5,16,17]. The reverse line blot hybridization assay (RLB) has been used to detect Mhf and CMhm in cats [18] however this assay has not been compared to other more sensitive molecular based methods.

Studies conducted to assess risk factors for feline haemoplasma infection, suggest that cats infected with FIV and/or FeLV may be more likely to harbour feline haemoplasmas and to have clinical signs associated with disease than non-infected cats [19-21]. Other studies however report no association between FIV and severity of clinical signs in haemoplasma infected cats [13,20,22-24]. Previous studies have shown that haemoplasma positive cats are more likely to be male [25-27] and that older male cats were more likely to be positive for feline haemoplasmas than younger or female cats [4,5].

The aims of this study were to compare the results of qPCR and RLB in the detection of feline haemoplasmas in naturally infected cats, and to determine if any association exists between infection and possible risk factors for disease such as age, gender and retrovirus status in the study population.

## Methods

### Sample collection, processing & DNA extraction

EDTA blood samples from cats included in this study were obtained from the following sources (i) Samples submitted to the Clinical Pathology Laboratory at the School of Veterinary Medicine (SVM) at the University of the West Indies (UWI), Trinidad (any repeat submissions were excluded) (ii) Trinidad and Tobago Society for the Prevention of Cruelty to Animals (TTSPCA) samples obtained when cats were euthanized or undergoing neutering for adoption purposes (iii) and a group termed ‘other’ which were from cats whose owners voluntarily presented their pets for the study.

Cats that appeared normal on clinical examination (i.e. bright, alert, responsive, normal appetite, no abnormal discharges and normal vital parameters) were classified as healthy whilst other cats were classified as not healthy. Written and/or verbal consent were received by all cat owners.

Data, when available, on age, gender and FeLV and FIV status (Snap® FIV/FeLV Combo Test, IDEXX Laboratories, Inc., Westbrook, ME) were obtained for each cat. Complete blood counts (CBC) were done for each sample within 48 hours of collection using an automated analyser (Sysmex K-4500 Haematology Analyzer, Sysmex America Inc., Mundelein, IL). Blood smears were prepared, stained with Wrights-Giemsa and examined microscopically for evidence of haemoplasmas.

DNA was extracted from 100 μL EDTA blood using the DNeasy blood and tissue kit (Qiagen Sciences, MD, USA) according to the manufacturer’s instructions. Extracted DNA was stored at -20°C in AE elution buffer until further analysis.

### RLB

The RLB was performed as a two-step process. First, a generic PCR was performed on samples followed by a hybridization step in a miniblotter apparatus (MN45, Immunetics, Cambridge, MA) of amplified products onto a membrane (Biodine C membrane, Pall Inc,Palo Alto, CA,USA) to which oligonucleotide probes for CMhm and Mhf were previously covalently bonded (Table 1). The generic PCR employed forward and reverse primers to amplify an approximately 400 bp fragment of the bacterial 16S rRNA gene: 16S 25f (5’-AGAGTTTGATCMTGGCTCAG and 16S 519r (biotin-5’-GWATTACCGCGGCKGCTG) [28-30], (Sigma-Genosys, TX, USA), respectively. Five μL of target DNA was amplified in a 50 μL reaction volume using Sigma REDTaq^TM^ ReadyMix ^TM^ PCR reaction mix (Sigma St. Louis MO, USA) according to the manufacturer’s instructions. Positive control CMhm and Mhf DNA samples were supplied by S. Tasker. Five μL of molecular grade water (Sigma St. Louis MO, USA) was used as a negative control. The PCR was performed in a Techne thermal cycler (Techne, Cambridge, UK) using the following protocol; initial denaturation at 94°C for 10 min, followed by 35 cycles of 94°C for 30 sec, 55°C for 30 sec and 45 sec at 72°C with a final extension step of 5 min at 72°C and a final hold at 4°C.

**Table 1:** 5’- 3’ Sequences of oligonucleotide probes used for the RLB

**Oligonucleotide probe**	**5’- 3’ Sequence**	**GenBank Accession no.**
*'Candidatus* Mycoplasma haemominutum'	TTCGCGAGCAGAGAGGAG AAGGGAGCGTTCTGGGAAAC	JX002102
*Mycoplasma haemofelis*	ATGATTTAGCTTTTAAAGCCT	DQ1571556- DQ1571560

Application of oligonucleotide probes to the membrane and hybridization of PCR products were done as previously described [31], with the following adaptations: 40 μL of PCR product was applied to the membrane and the second post hybridization wash was done at 52°C to remove any non-specific products which may have hybridized onto the membrane.

### qPCR

Fifty μL of extracted DNA from each blood sample was shipped to the University of Bristol, UK for analysis for feline haemoplasmas by qPCR as described previously [16]. Briefly, five μL of target DNA was amplified separately for each of the three haemoplasma species with each qPCR duplexed with an assay to detect feline 28S rDNA as an internal control.

### Statistical analysis

A haemoplasma positive cat was defined as one which had a positive feline haemoplasma result. The qPCR assay was used as the gold standard for a positive feline haemoplasma result for the purpose of statistical analysis of risk factors. The Mann-Whitney U test (M-W) was used to test for differences between the feline haemoplasma positive and negative cats with respect to the non-normally distributed continuous variables age and haematocrit (HCT). Association between haemoplasma infections and discrete variables (gender, FeLV/FIV status, anaemic (HCT < 0.24 L/L) vs. non anaemic (HCT ≥ 0.25 L/L) status [32], were analyzed by the chi square test (χ^2^) with the continuity correction applied, and the Fisher exact test for cell frequencies of ≤ 5. Statistical significance was set at a P value < 0.05. Logistic regression was used to predict haemoplasma infections using predictor variables which may be associated risk factors for infection. Data were analyzed using SPSS version 12 (SPSS Inc., Chicago, IL) or MLwiN version 2.02 (Multilevel Models project Institute of Education). Test sensitivity, specificity, positive and negative predictive values were calculated using the programme Win Episcope vers 2 (Epidecon http://www.clive.ed.ac.uk/winepiscope/).

## Results

### Microscopic examination of blood smears

Haemoplasma organisms were not identified in any of the samples examined microscopically.

### qPCR and RLB results

A total of 154 blood samples were entered into the study. Two samples were removed from statistical data analysis as they were negative for feline 28S rDNA by qPCR. For each batch, the RLB detected the positive control CMhm and Mhf DNA supplied by S. Tasker, and the negative control was consistently negative.

For CMhm, 40 (26.3%) and 48 (31.6%) cats were positive by RLB and qPCR respectively. For Mhf, RLB identified 11 (7.2%) positive samples whilst qPCR detected 41 (27.0%) positive samples. CMt was detected in three samples by qPCR. Mixed haemoplasma infections were detected in five samples by RLB and in 19 samples by qPCR (Table 2). The qPCR detected 16 samples with both CMhm and Mhf and of these, the RLB detected four with both CMhm and Mhf, nine with CMhm only, one with Mhf only and two were negative. Of the two which were positive by qPCR for coinfections of CMhm, Mhf and CMt, RLB detected CMhm and Mhf in one and CMhm only in the other. RLB detected CMhm in one sample that was coinfected with CMhm and CMt. Of the 23 samples positive by qPCR for Mhf only, RLB detected five. Finally, RLB detected 24 of the 29 samples that were positive for CMhm only by qPCR. All samples that were negative using qPCR were also negative by RLB.

**Table 2:** Comparison of the RLB and qPCR assays for feline haemoplasmas (N =152)

**Result**	**qPCR result No. (%)**	**RLB result No. (%)**
CMhm only	29 (19.1)	35 (23.0)
Mhf only	23 (15.1)	6 (4.0)
CMhm and Mhf only	16 (10.5)	5 (3.3)
CMhm and CMt	1 (0.7)	0
CMhm, Mhf and CMt	2 (1.3)	0
Negative	81 (53.3)	106 (69.7)
Overall positive	71 (46.7)	46 (30.3)

CMhm = ‘*Candidatus* Mycoplasma haemominutum’.

Mhf = *Mycoplasma haemofelis*.

CMt = ‘*Candidatus* Mycoplasma turicensis’.

Using qPCR as a gold standard, the sensitivity of RLB in detecting any haemoplasma infection was 64.7%, for CMhm detection 83.3%, and for Mhf detection 26.8%. The specificity and the positive predictive value of the RLB for the feline haemoplasmas was 100.0%. The sensitivity, specificity, positive and negative predictive values and their 95% confidence intervals are displayed in Table 3.

**Table 3:** Sensitivity, specificity, positive and negative predictive values (%) and 95% confidence intervals (CI) of RLB for the detection of feline haemoplasmas using qPCR as the gold standard

**Parameter**	**Sensitivity <95%CI>**	**Specificity**	**Positive Predictive value**	**Negative Predictive value <95%CI>**
Positive for any haemoplasma	64.7 <53.7-75.9>	100.0	100.0	76.4 <68.3-84.5>
‘*Candidatus* Mycoplasma haemominutum’	83.3 <72.8-93.9>	100.0	100.0	92.9 <88.1-97.6>
*Mycoplasma haemofelis*	26.8 <13.3-40.4>	100.0	100.0	78.7 <72.0-85.5>

### RLB and qPCR copy numbers/100μL blood for a positive result for CMhm and Mhf

qPCR threshold cycle (C_t_) values for Mhf ranged from 18.6 to 42.4 whilst CMhm and CMt C_t_ values ranged from 15.8 to 41.1 and 35.7 to 39.2 respectively. Feline 28S rDNA C_t_ ranged from 25.0 to 40.9 in these reactions.

**Figure 1:**
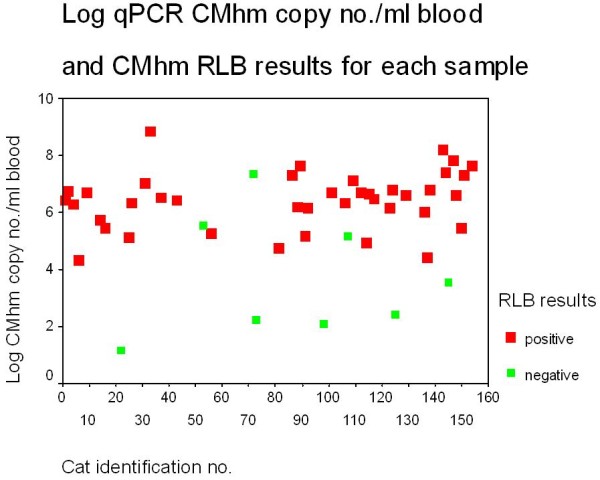
**Log qPCR CMhm copy no. /100μL blood and CMhm RLB results for each sample.** For a positive RLB CMhm result, the highest and lowest CMhm copy no./100μL blood as determined by qPCR were 6.9 x 10^8^ (log_10_ = 8.8) and 2039 (log_10_ = 4.3) copy no./100μL blood and for a negative RLB CMhm result, 2.2 x 10^7^ (log_10_ = 7.35) and 13.8 (log_10_ = 1.1) copy no/100μL blood. Eight CMhm samples were positive by qPCR but negative by RLB.

Calculation of copy numbers (copy no.) for positive samples was done as described previously. For a positive RLB CMhm result, the highest and lowest CMhm load as determined by qPCR were 6.9 x 10^8^ (log_10_ = 8.8) and 2039 (log_10_ = 4.3) copy no./100μL blood and for a negative RLB CMhm result, 2.2 x 10^7^ (log_10_ = 7.35) and 13.8 (log_10_ = 1.1) copy no/100μL blood. These data are displayed in Figure 1.

Eight CMhm samples were positive by qPCR but negative by RLB. The quantity of CMhm for these RLB negative samples was less than 20,239 (log_10_ = 4.31) copy no./100μL of blood for 5/8 discordant samples and the highest quantity of CMhm recorded in the other 3 samples was 2.2 x 10^7^ (log_10_ = 7.35) copy no/100μL blood. Overall, only 10.4% of samples which were positive for CMhm by qPCR, had a CMhm load of less than 20,239 (log_10_ = 4.31) copy no/100μL blood.

**Figure 2:**
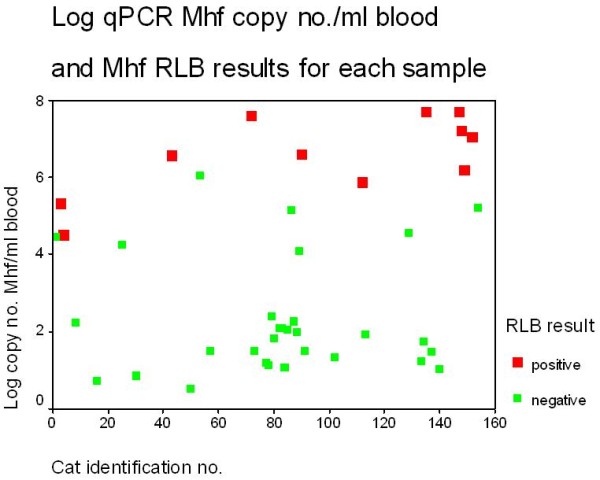
**Log qPCR Mhf copy no. /100μL blood and Mhf RLB results for each sample.** For a positive RLB Mhf result, the highest and lowest Mhf copy no./100μL blood as determined by qPCR were 5.0 x 10^7^ (log_10_ = 7.7 ) and 32,457 (log_10_ = 4.5) and for a negative RLB Mhf result 1.1 x 10^6^ (log_10_ = 6.05) and 3.4 (log_10_ = 0.53), copy no./100μL blood. Thirty Mhf samples were positive by qPCR but negative by RLB.

For a positive RLB Mhf result, the highest and lowest copy no./100ÂµL blood according to the qPCR for Mhf were 5.0 x 10^7^ (log_10_ = 7.7 ) and 32,457 (log_10_ = 4.5) respectively. For qPCR positive Mhf but negative RLB Mhf samples, the highest and lowest Mhf loads were 1.1 x 10^6^ (log_10_ = 6.05) and 3.4 (log_10_ = 0.53), copy no./100μL blood respectively. These results are displayed in Figures 2. Overall, 56.1% of Mhf positive samples had Mhf loads of below 254 (log_10_ = 2.4) copy no./100μL blood.

The quantity of CMt in the three samples that were positive by qPCR was 133 (log_10_ = 0.82), 996.4 (log_10_ = 1.7) and 109 (log_10_ = 0.7), copy no/100μL respectively.

### Case characteristics

Out of 152 samples that were positive for the internal control feline 28S rDNA , 45 (29.6%) cats came from the TTSPCA, 54 (35.5%) cats were patients at the SVM, whilst 53 (34.9%) were obtained from other sources. Age was known for 109 cats, with ages ranging from 2 months to 20.0 years (median 2.0 years). The gender of 139 cats was recorded: 80 (57.6%) were male and 59 (42.4%) were female. The retrovirus status was known for 138 cats. Overall 24 (15.8%) were retrovirus positive and 114 (75.0%) were negative: Of the cats that were retrovirus positive, 3 were positive for both FeLV and FIV, 12 for FeLV only and 9 for FIV only.

The overall health status of 117 cats was determined: 67 (57.3%) cats appeared healthy and 50 (42.7%) were not healthy. There was a significant association between source of samples and health status, (χ^2^ , P < 0.01); the majority of cats classified as healthy were from the category “other” (32, 47.8%) and those classified as not healthy from the SVM laboratory (38, 76.0%), (Table 4).

**Table 4:** Classification of study population by source of sample and health status (N = 117)

**Source**^**1**^	**Health status**	
	**Not healthy (No. %)**	**Healthy (No. %)**
SVM Clinic	38 (76.0)	8 (11.9)
TTSPCA	2 (4.0)	27 (40.3)
“Other”	10 (20.0)	32 (47.8)

^1^TTSPCA = Trinidad and Tobago Society for the Prevention of Cruelty to Animals, SVM = School of Veterinary Medicine, “Other” includes voluntary submission by pet owners.

### Risk factors for feline haemoplasma infection

Cats that were haemoplasma positive were significantly older (median age 5.5 years, interval 4 months – 15 years) than non-infected cats (median age 1.5 years, interval 2 months -19.5 years, (M-W, P < 0.01). Age was also significantly associated with CMhm infections (M-W, P < 0.01) and Mhf infections (M-W, P = 0.03).

Gender was found to be significantly associated with CMhm status (χ^2^, P = 0.03). However, this study found no significant association for gender and overall haemoplasma status (χ^2^, P = 0.06) and Mhf status (χ^2^ P = 0.89).

Retrovirus status was found to be significantly associated with haemoplasma status (χ^2^, P = 0.01). In addition, CMhm was significantly associated with both FIV (χ^2^, P < 0.01) and FeLV (χ^2^, P < 0.01) infections. This study found no significant associations between any retrovirus and Mhf status (χ^2^, P > 0.05).

Health and anaemia status were not significant predictors for haemoplasma status (χ^2^, P > 0.05). HCT values were known for 145 samples and of these, seven cats were anaemic. Two anaemic cats were positive for CMhm, Mhf and FeLV.

### Logistic regression model

Two logistic regression analyses were performed to model the overall probability of (a) haemoplasma status and (b) CMhm status as outcome variables and three risk factors as predictors: age, retrovirus status and gender. For both models, a test for the full model with all three predictors against a constant only model was significantly different (P < 0.01). Age was a significant predictor for an overall positive haemoplasma result, odds ratio (OR) = 1.10 (95% C.I (1.01-1.25, P = 0.02). Retrovirus status was also a significant predictor for an overall positive haemoplasma result. Retrovirus positive cats were 5.68 (95% CI 1.35-21.2, P = 0.02) times more likely to have a positive haemoplasma result. Gender was not a significant predictor for haemoplasma status when modelled with age and retrovirus status (Table 5). Retrovirus status, gender and age were all significant predictors for a positive CMhm result. Cats that were retrovirus positive were 8.8 (95% C.I 2.51-30.8, P < 0.01) times more likely to have a positive CMhm result and male cats were 3.4 (95% CI 1.06- 10.66, P = 0.04) times more likely than females to have a positive CMhm result. The odds of CMhm infections increased by 1.16 (95% CI 1.03-1.30, P = 0.01) for each one year increase in age (Table 6).

**Table 5:** Relationship between predictor variables and any haemoplasma qPCR positive result using logistic regression analysis

	**Regression coefficient**	**S.E**	**Odds ratio**	**95% C.I**	**P value**
Retrovirus status					
(positive vs negative)	1.67	0.70	5.68	1.35 - 21.20	0.02
Gender (male vs female)	0.85	0.47	2.30	0.96 **-** 6.00	0.07
Age (years)	0.12	0.05	1.10 ^1^	1.01 - 1.25	0.02
Constant	−1.34	0.40			<0.02

^1^ Odds ratio per 1 year increase in age.

**Table 6:** **Logistic regression model for predictor variables and qPCR positive results for ‘*Candidatus*Mycoplasma haemominutum’**

**Variable**	**Regression coefficient**	**SE**	**Odds ratio**	**95% CI**	**P value**
Retrovirus status	2.18	0.64	8.80	2.51 - 30.80	< 0.01
(positive vs negative)					
Gender (male vs female)	1.21	0.58	3.40	1.06 - 10.66	0.04
Age (years)	0.15	0.06	1.16 ^1^	1.03 - 1.30	0.01
Constant	−2.89	0.60			< 0.01

## Discussion

This is the first report of a comparison of the RLB and qPCR in the detection of feline haemoplasmas. It is important to note that the RLB and qPCR were in complete agreement for all samples that were negative by qPCR. The discordant results were observed in samples with both low and high haemoplasma copy numbers and also, in general, with Mhf positive samples. This could be explained by the fact that the RLB is a two-step process of a non-specific PCR followed by a hybridization procedure, hence there are a number of variables which may lead to a lack of sensitivity at each step. Such factors include the competition for substrate in PCR reactions and different hybridization temperatures for amplified DNA during the RLB. The qPCR detected 19 cats with mixed infections whilst the RLB detected only five. The RLB detected CMhm more often in these samples.

The majority of the Mhf positive samples in this study had apparently very low concentrations of Mhf DNA compared to samples that were positive for CMhm. This factor may therefore account for the large discrepancy when comparing RLB and qPCR results for Mhf and CMhm. DNA standards were not used to compare RLB and qPCR data and using standard dilutions of Mhf and CMhm DNA would provide a more definite indication on the empirical level of detection of these haemoplasmas using the RLB. The results from this study indicate that the RLB is limited in its ability to detect Mhf.

From a clinical perspective, cats with very low copy nos. of feline haemoplasma DNA may not show any significant clinical signs of disease.

Concerning risk factors for haemoplasma infections, previous studies, have shown that haemoplasma positive cats were more likely to be male [11,25,27]. Although more males had an overall positive haemoplasma result than females in the present study, this difference was not statistically significant. Gender was however significantly associated with a positive CMhm status but not with a positive Mhf status.

This study found that older cats were significantly more likely to be haemoplasma positive than younger cats, which is in agreement with previous studies [4,27]. Studies conducted in Australia and the United Kingdom found that older male cats were more likely to be positive for feline haemoplasmas than younger or female cats [4,27,33]. A study to detect CMhm and Mhf conducted in Germany on 262 cats using cPCR and restriction fragment length polymorphism (RFLP) on positive samples, found that male cats were more likely to be infected with CMhm than female cats and that CMhm infections were also significantly associated with FeLV and FIV infections [13]. The present study supports previous findings as CMhm infections were found to be significantly associated with gender, retrovirus status and age. The higher prevalence of haemoplasma infections observed in male cats in the present study may be due in part to their behaviour characteristics where they are more prone to roam and encounter the flea vector or acquire the agent if fighting with other cats.

Results of this study demonstrate a significant association between feline haemoplasma infection and retrovirus status. There was also a significant association between FeLV, FIV and CMhm status. This is in agreement with a study on feral cats in Northern Florida [34], which showed a significant association between FIV and FeLV infection and the presence of CMhm. However, in contrast to a study conducted in the USA on 310 cats with cytological evidence of haemoplasmosis [25], the present study found no significant association between Mhf and FIV status. The present study is also in agreement with a study conducted in Brazil which found that cats with FIV and FeLV were more likely to be haemoplasma positive, (mainly due to CMhm) than retrovirus negative cats [20].

Many cats in this study population that were haemoplasma positive were clinically normal and there was no significant association between the presence of CMhm or Mhf DNA and health status. A similar finding was observed in a Swiss study [27]. The extent to which infections with the haemoplasmas contributed to clinical illness was not known for the present study as many cats were infected with the feline retroviruses and also presented with signs of systemic diseases. It is however important to identify cats that are subclinically infected with the feline haemoplasmas as concurrent immunosuppression may lead to illness [35].

## Conclusions

qPCR was more sensitive than the RLB in detecting the feline haemoplasmas however the RLB may be used to screen and differentiate between the CMhm and Mhf in cats with potentially high to moderate bacteraemia. A negative RLB result may be a false negative however a positive result is indicative of infection.

Age (> 5.5 years) and retrovirus positivity appear to be common risk factors for infection with the feline haemoplasmas. Further studies on feline haemoplasma infections in cats are needed to determine the significance of detecting small amounts of haemoplasma DNA, feline retrovirus infection and other associated risk factors on the clinical manifestation of disease.

## Competing interests

The authors declare that they have no competing interests.

## Authors’ contributions

KG authored the manuscript, designed the study, conducted the RLB and analysed the data. CE assisted in study design, interpretation of data and edited the manuscript. TA, NS and AP assisted with sample collection, haematology and retrovirus testing and managing the database. OS assisted in designing the oligonucleotide probes and editing the manuscript. ST performed the qPCR and assisted in drafting and editing the manuscript. The authors declare that there are no conflicts of interest. All authors read and approved the final manuscript.
